# Effects of a 12-week training programme on selected hormonal and psychological parameters and their interrelationships in highly-trained male and female swimmers

**DOI:** 10.5114/biolsport.2025.145910

**Published:** 2024-12-13

**Authors:** Olga Surała, Jadwiga Malczewska-Lenczowska, Dariusz Turowski, Paweł Słomiński, Maciej Certa, Blair T Crewther

**Affiliations:** 1 Institute of Sport – National Research Institute, Warsaw, Poland; 2 Józef Piłsudski University of Physical Education, Warsaw, Poland; 3 School of Science and Technology, University of New England, Armidale, Australia

**Keywords:** Recovery, Endocrinology, Anxiety, Overtraining, Tapering

## Abstract

Swimming training load (TL) is regarded as a major stimulus for hormonal adaptation, but research findings are inconsistent. Methodological limitations also exist (e.g., cross-sectional sampling) with little knowledge of acute hormone responses and hormone-psychological interactions that regulate training outputs. These issues were addressed in a 12-week training study on highly-trained swimmers. Eighteen swimmers (10 males, 8 females) completed a 12-week training programme, involving a stepwise reduction in TL before a major competition. Testing was conducted from Monday–Friday at week one (T1), week five (T2), and week 12 (T3), including measures of salivary testosterone and cortisol, willingness to train, stress, and sleep quality. Post-session hormones were assessed on Mondays and Fridays. Daily-averaged swimming distance decreased by -21% (T2) and -57% (T3), relative to T1 (p < 0.001). We found no significant training effect on the cortisol and testosterone measures, both baseline and acute exercise response, willingness to train, and sleep quality. Only stress varied with training, decreasing significantly at T2 and T3 from T1 in female swimmers. Among male swimmers, daily changes in baseline cortisol and testosterone were related (r = 0.45) at T1, as was sleep quality and stress (r = 0.39) at T3. In summary, highly-trained swimmers showed little or no adaptive changes prior to a major competition. The one exception was self-reported stress among female swimmers, which decreased along with TL. The emergence of daily interrelationships (in male swimmers only) between selected hormonal or psychological outputs could provide a new lens to assess pre-training preparation.

## INTRODUCTION

Swimming is a popular sport with a diverse range of technical strokes (i.e., breaststroke, butterfly, freestyle, backstroke) and distances (e.g., 50 m to 1500 m) in an Olympic swimming programme, with openwater events exceeding 10 km or more [1]. Training load (TL) is a key concept in swimming, with distances swum by trained individuals ranging from 1.8–16 km/day [2, 3, 4, 5]. Exposure to different TLs can promote hormonal adaptations [6]. Studies have reported a rise in baseline cortisol and/or testosterone concentrations with a reduction in swimming TL [2, 4, 7], as adaptive mechanisms to support performance and recovery. Nevertheless, a review of evidence revealed inconsistent hormonal responses [6], whilst no experimental changes in cortisol or testosterone concentration were seen with varying TLs [8, 9, 10, 11], making it difficult to ascertain the main driver/s of this adaptive pathway.

A closer inspection of this research reveals some methodological limitations. As one example, studies often take a “cross-sectional” approach at each measurement occasion and this makes it harder to detect a real change, due to training, from natural daily variability. To highlight this point, one study demonstrated that concentration measures of salivary cortisol and testosterone in adolescent swimmers varied (over 7 consecutive days) by 27–38% across control and competition weeks [12]. Consequently, repeated tests over several days are needed to decrease uncertainty in the modeled values and establish a true training effect. Moreover, whilst baseline hormone activity has been well studied among swimmers [4, 7, 8, 9, 10, 11], few have investigated the acute hormone response to exercise [4, 13]. This adaptive pathway has some discriminative value and it offers a functional index of genetic differences in stress reactivity [14]; a potential limiting factor in sport and training adaptation.

Traditionally, attempts to link baseline hormones to swimming adaptation have focused on endpoint outcomes (e.g., performance changes) [9, 15], rather than daily training outputs that mediate adaptive changes. This includes psychological processes (e.g., motivation to train, stress, sleep quality, recovery) linked to daily changes or differences in athlete cortisol and/or testosterone levels [7, 16, 17, 18]. Such a viewpoint is coherent with the shortterm effects of endogenous hormones on human performance. That is, cortisol and testosterone both exert rapid actions on the neuromuscular system that can manifest in seconds (e.g., second messengers, neuron activation) or hours (e.g., behavior, cognition) [14]. Another drawback of literature lies in the common practice of pooling data from male and female swimmers [4, 8, 10, 12, 19, 20], thereby preventing a closer investigation of sex-related differences or similarities. Addressing these issues would improve the veracity of research findings and expand current knowledge, especially with the ecological testing of swimmers.

This study investigated the hormonal (i.e., testosterone, cortisol) and psychological (i.e., willingness to train, stress level, sleep quality) responses of highly-trained male and female swimmers to a 12-week training programme, involving a stepwise reduction in TL prior to a major competition. Tests were conducted at the start (T1), middle (T2), and end (T3) of this period. Our first hypothesis was that some hormonal changes (i.e., increase in baseline cortisol and testosterone levels) would occur from T1 to T3, coinciding with less stress and better recovery (i.e., improved willingness to train and sleep quality). Our second hypothesis was that daily interrelationships between selected hormonal and psychological variables would emerge (i.e., testosterone and willingness to train, cortisol and stress level).

## MATERIALS AND METHODS

### Participants

Eighteen highly-trained swimmers, who were guided by the same coaching team, were recruited for this study. This sample comprised of 10 males (mean ± SD; age 23.3 ± 2.6 years, height = 184.5 ± 3.7 cm, body mass = 78.0 ± 3.6 kg, training experience = 13 ± 3 years) and eight females (age 17.6 ± 2.2 years, height = 171.5 ± 4.5 cm, body mass = 64.7 ± 7.9 kg, training experience = 8 ± 2 years). Event specialization included all four technical strokes (i.e., 1 butterfly, 5 breast stroke, 7 freestyle, 5 backstroke) and swimming distances that varied from 50 m to 800 m. Exclusion criteria included use of any doping substances, a major injury likely to limit training, any medical or hormone-related disorder, and recent travel across different time zones prior to testing. Written informed consent was given after subjects were briefed on the study design, risks, and benefits. Approval was also obtained from a parent, or guardian, for any athlete of non-consenting age (< 18 years). This study is part of a larger project [4] that received ethical approval (KEBN-19-43-OS) from the Institute of Sport – National Research Institute, Poland. Here we present unpublished data with some conceptual and methodological differences from the published article [4] to form a standalone paper.

### Study design

The participants were monitored across a 12-week training period, in preparation for the Polish National Swimming Championships. This event was deemed critical to obtaining qualifying times (and selection) for the 18^th^ FINA World Championships and/or European Junior Swimming Championships later in the year. Testing began at week one (T1), being the start of the general preparation phase, with retesting at week five (T2) at the start of the specific preparation phase. The final test was implemented at week 12 (T3), representing completion of the pre-competition tapering cycle. Testing at T3 ended three days before the national event commenced. Replicate measures of salivary testosterone and cortisol, both baseline and acute exercise-induced responses, and psychological state (i.e., willingness to train, stress, sleep quality) were taken using a daily testing procedure at T1–T3; see below for more details.

### Training plan

A traditional periodization plan was followed to ensure the athletes were in peak physical condition for the Polish National Swimming Championships [9, 21, 22, 23]. This plan involved a progressive decrease in TL, as evidenced by the total swimming distance completed over a typical training week: T1 = 66.7 km (95% confidence interval [CI] 63.4, 70.8), T2 = 54.6 km (95% CI 51.9, 57.4), and T3 = 28.2 (95% CI 26.8, 29.7) [4]. Subsequently, a concomitant decrease in the total amount of time spent training (in the pool) was observed: T1 = 19.6 hrs (95% CI 18.1, 21.0), T2 = 18.5 hrs (95% CI 17.0, 19.8), and T3 = 10.3 (95% CI 9.5, 11.1). The distribution of TL within a week was commensurate with standard practices. Specifically, the highest volume of swim-based training was prescribed on Monday and Tuesday, with a gradual reduction in training load from Wednesday to Saturday. Sunday was the allocated rest day for all swimmers.

**FIG. 1:**
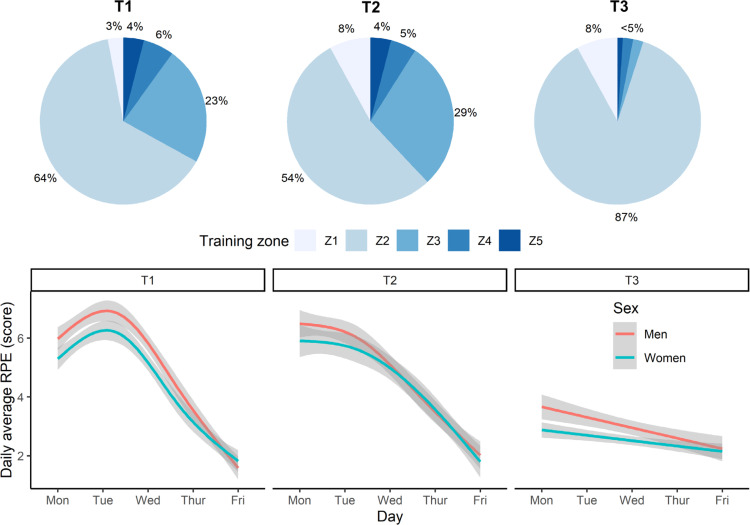
Swim-training intensity at the start (T1), middle (T2), and end (T3) of the 12-week training programme. Top plot shows the percentage of training performed at different intensity zones: Z1 (blood lactate < 1 mmol/l; skills), Z2 (1–3 mmol/l; aerobic), Z3 (3–5 mmol/l; aerobic), Z4 (> 5 mmol/l; anaerobic), and Z5 (maximal effort or sprint training). Bottom plot depicts the daily-averaged rating of perceived exertion (RPE) across the AM and PM swim training sessions.

The intensity of training varied according to conditioning and recovery goals. This was achieved by prescribing one of five training-intensity zones, based on blood lactate profiles taken before this study: Z1 (< 1 mmol/l; skills work), Z2 (1–3 mmol/l; aerobic conditioning), Z3 (3–5 mmol/l; aerobic conditioning), Z4 (> 5 mmol/l; anaerobic conditioning), and Z5 (maximal effort or sprint training) [24]. Physical effort or intensity was gauged subjectively, using Likert-scored (1–10 range) ratings of perceived exertion (RPE) collected after each swimming session. The dailyaveraged RPE trajectories paralleled that of training volume; falling across a training week and generally decreasing from T1 to T3. Daily-averaged swimming distance and RPE were also strongly related (*r* = 0.65). Given these similarities, we limit our focus to the training volume component. Figure 1 illustrates the different training zones and the smoothed trajectories for RPE within and between training weeks.

Weekly training frequency was maintained at T1, T2 and T3, comprising of six early-morning pool sessions each lasting up to two hours (starting at 6:00 am at T1 and T2, and 6:45 am at T3) and five afternoon pool sessions (scheduled from 4:00–6:00 pm). On average, around 52% of daily swimming distance was completed in the morning session, which indicates that training was evenly distributed across AM and PM sessions. A “light” pool session was completed on Saturday morning, lasting no more than one hour. Two “dry-land” workouts were also performed each week, immediately after a morning pool session.

### Assessment procedures

Participant testing took place over five consecutive days (Monday– Friday), at each time point (T1–T3), for a total of 15 scheduled tests. Data were collected at the training venue before and/or after the early-morning pool session. Upon arrival at the training facility, each participant first rated their willingness to train, stress level, and sleep quality on a 1–5 Likert scale (anchored from 1 = was very poor up to 5 = very good). Next, the participant provided a pre-session (or baseline) saliva sample that was collected by passive drool into a pre-labelled 5 mL polypropylene tube. These samples were taken 1.01 ± 0.35 hrs after waking, based on self-reported wake times, which avoided bias associated with the early morning surge in cortisol [25]. Post-session samples (Mondays and Fridays only) were collected ~10 minutes after exercise completion, so that a hormonal change score can be calculated to index the acute hormone response to exercise. Given the ecological design and fixed training plan, it was not possible to increase rest periods (e.g., 2 days or more) prior to data collection, apart from early-morning testing on Mondays at T1, T2, and T3.

The subjects maintained their normal dietary habits, which generally included a light breakfast, fruit juice and/or caffeine (e.g., coffee) prior to training. To prevent sample contamination, the swimmers were asked to restrict food or fluid intake at least 30 minutes before sampling. All samples were stored on ice and returned to the laboratory by 10:00 am on each testing day, before storage in a -80° C freezer. After thawing and centrifugation, the samples were assayed in duplicate for testosterone and cortisol concentrations using enzyme-linked immunoassay kits (DRG, Germany). Inter-assay variability on low and high controls, expressed as a coefficient of variation (CV), did not exceed 10% in each plate. Each participants’ samples were assayed in the same plate to eliminate this source of variation.

### Statistical analyses

The data were analyzed using R software [26]. Some hormonal and psychological data (1–3% of total) were missing due to illness, work, and study commitments. Missing data were imputed by linear interpolation at the person level. Prior to testing, testosterone and cortisol were log-transformed to normalize distribution and eliminate nonuniformity bias [4]. To evaluate training efficacy, each variable was examined using a two-way (i.e., time [3 levels], sex [2 levels]) analysis of variance with repeated measures. Models were constructed in the lmerTest package [35]. Selected covariates (e.g., day, sex, time since waking, weekly TL distribution) were also tested and retained in a model if they influenced, significantly, the dependent variable. Observed power in each model was determined using partial eta squared (η^2^). Where appropriate, post-hoc contrasts were performed with the Tukey test and Cohen’s *d* calculated as an effectsize statistic. The *d* scores are interpretable as a small (0.2 to < 0.5), medium (0.5 to < 0.8), large (0.8 to < 1.2), very large (1.2 to < 2.0) or huge (2.0+) effect [27]. Modeled data are presented as estimated marginal means with a 95% CI.

To test for daily interrelationships between study variables, we ran a series of multilevel correlations (separately for men and women at T1, T2 and T3) using the correlation package [28]. Multilevel correlations are a special case of correlations, where a factor (i.e., each participant) was included as a random effect in a mixed-effects model [28]. Consequently, the *r* values represent within-person, pairwise relationships. Being regression based, each multilevel relationship also signifies a partial correlation that controls for all other variables. The partial *r* values were interpreted as weak (*r* = 0.20 to < 0.40), moderate (*r* = 0.40 to < 0.60), strong (*r* = 0.60 to < 0.80) or very strong (*r* = 0.80 to 1.00) effects [29]. To account for multiple comparisons, the *p* values were adjusted using a false-discovery procedure. Statistical significance for all analyses was set at *p* < 0.05.

## RESULTS

**FIG. 2:**
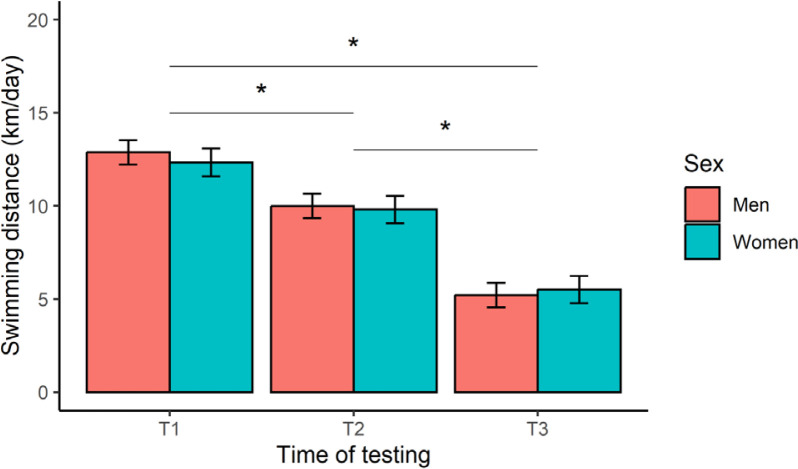
Marginal means (error bars represent the 95% CI) for daily-averaged swimming distance at the start (T1), middle (T2), and end (T3) of the 12-week training programme. *Significant difference between test occasions.

The 12-week training programme was deemed successful, based on a significant (*p* < 0.001) improvement in registered mean FINA points from T2 (673 ± 96 points) to T3 (720 ± 109 points) for all swimmers, representing a very large effect (*d* = 1.2). Insufficient swimming data were available to compute FINA points at T1. Our main analyses yielded a significant time effect on daily-averaged swimming distance (F _[2, 244]_ = 270.6, *p* < 0.001, η^2^ = 0.69), after controlling for TL distribution within a week (Figure 2). Post-hoc contrasts confirmed a stepwise reduction in TL, with a greater (*p* < 0.001, large or very large effects) swimming distance achieved at T1 compared to T2 (*d* = 1.1) and T3 (*d* = 2.9), and at T2 versus T3 (*d* = 1.8). Expressed as a percentage of T1, these results represent a TL reduction of -21% (T2) and -57% (T3). No significant effect of sex (F _[1, 16]_ = 0.18, *p* = 0.678, η^2^ = 0.01), or a sex × time interaction (F _[2, 244]_ = 0.91, *p* = 0.403, η^2^ = 0.01), was observed for this outcome.

**FIG. 3:**
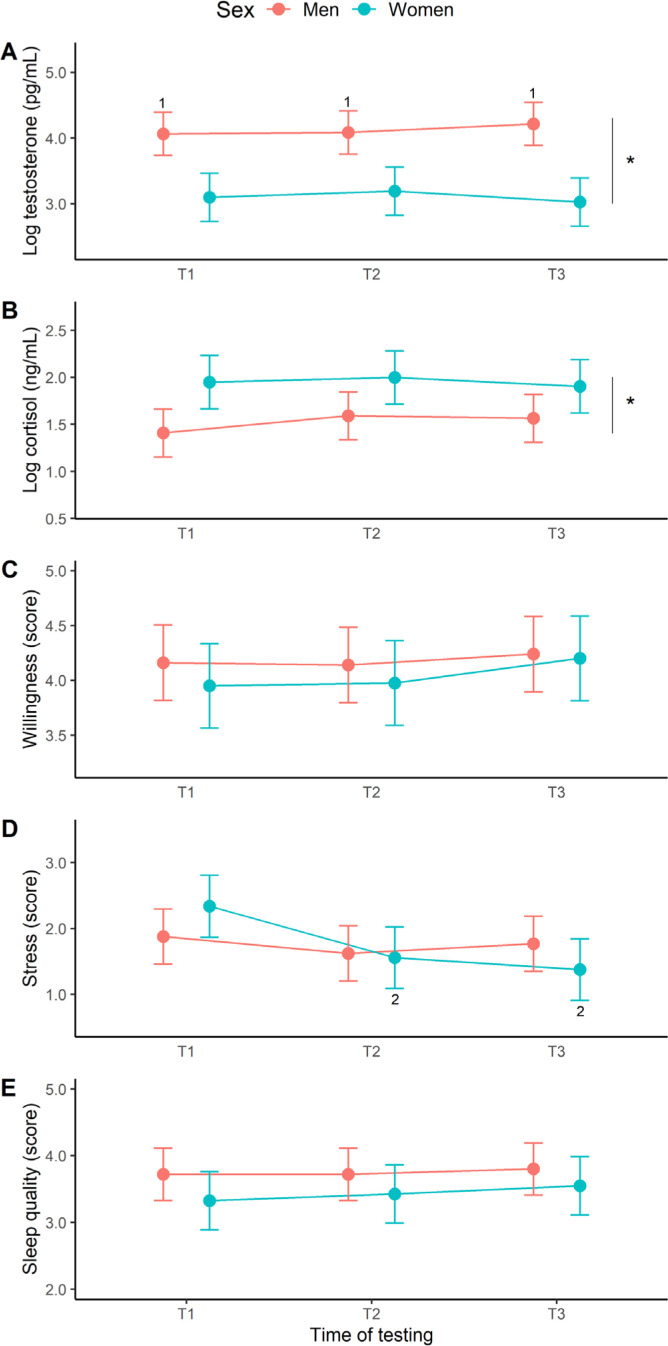
Marginal means (error bars represent the 95% CI) for the baseline hormonal and psychological measures at the start (T1), middle (T2), and end (T3) of the 12-week training programme. 1Significantly different from women at T1, T2 and T3, 2Significantly different from women at T1, *Significant main effect of sex.

The baseline results are depicted in Figure 3. For testosterone (Figure 3A), a sex effect (F _[1, 16]_ = 19.71, *p* < 0.001, η^2^ = 0.55) and sex × time interaction (F _[2, 248]_ = 6.66, *p* = 0.002, η^2^ = 0.05) was identified, but no time effect (F _[2, 248]_ = 1.02, *p* = 0.363, η^2^ = 0.01). Post-hoc analyses revealed significantly higher testosterone levels in male than female swimmers at all time points (*d* = 1.8–2.4, very large effects). For cortisol (Figure 3B), a sex effect emerged (F _[1, 16]_ = 6.47, *p* = 0.022, η^2^ = 0.29) with women presenting a higher concentration overall than men (*d* = 1.3), but no time effect (F _[2, 247]_ = 1.74, *p* = 0.178, η^2^ = 0.01) or interaction (F _[2, 247]_ = 1.35, *p* = 0.261, η^2^ = 0.01). Our assessment of willingness to train (Figure 3C) revealed no time (F _[2, 248]_ = 1.91, *p* = 0.150, η^2^ = 0.02), sex (F _[1, 16]_ = 0.38, *p* = 0.547, η^2^ = 0.02) or interactive effects (F _[2, 248]_ = 0.41, *p* = 0.661, η^2^ = 0.00). Perceived stress (Figure 3D) did vary with respect to time (F _[2, 247]_ = 18.83, *p* < 0.001, η^2^ = 0.13), but not sex (F _[1, 16]_ = 0.00, *p* = 0.998, η^2^ = 0.00), with a significant interaction (F _[2, 247]_ = 9.35, *p* < 0.001, η^2^ = 0.07). Follow-up tests identified a significant decline in female stress scores from T1 to T2 (*d* = -0.7, medium effect) and T3 (*d* = -0.8, large effect). The effects of time (F _[2, 248]_ = 1.11, *p* = 0.332, η^2^ = 0.09), sex (F _[1, 16]_ = 1.49, *p* = 0.240, η^2^ = 0.01), and their interplay (F _[2, 248]_ = 0.25, *p* = 0.777, η^2^ = 0.01), on sleep quality (Figure 3E) were all non-significant.

**FIG. 4:**
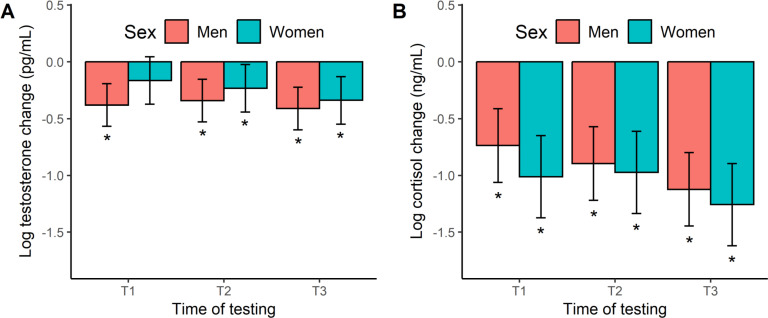
Marginal means (error bars represent the 95% CI) for the acute hormonal response to exercise at the start (T1), middle (T2), and end (T3) of the 12-week training programme. *Significant hormonal change from baseline (i.e., 95% CI excludes zero).

Figure 4 shows the acute hormone responses to exercise. The testosterone change scores in men and women (Figure 4A) were all negative and largely significant across exercise, based on a 95% CI that excluded zero. The one exception being female swimmers at T1. These responses equate to percentage changes of -21% to -34%. When modelling the acute testosterone response, we found no significant effect of time (F_[2, 86]_ = 0.77, *p* = 0.468, η^2^ = 0.02), sex (F_[1, 16]_ = 1.93, *p* = 0.183, η^2^ = 0.11), or a time × sex interaction (F_[2, 86]_ = 0.35, *p* = 0.706, η^2^ = 0.01). Like testosterone, the cortisol change scores (Figure 4B) were negative in both men and women, and significantly so at all time points, ranging from a -52% to -72% decline. Our modeling of the acute cortisol response did not reveal any significant effects with regards to time (F_[2, 86]_ = 1.88, *p* = 0.158, η^2^ = 0.01), sex (F_[1, 16]_ = 1.33, *p* = 0.266, η^2^ = 0.01), and their interplay (F_[2, 86]_ = 0.17, *p* = 0.840, η^2^ = 0.00).

The multilevel correlations are displayed in Table 1. Significant within-person relationships were sparse and limited to male swimmers at T1, between baseline cortisol and testosterone concentrations (small positive effect), and at T3, between sleep quality and stress (small positive effect). As a follow-up procedure, we added the RPE scores following AM training sessions, but this variable was unrelated to any morning-based measurement. Finally, we entered daily swimming distance as a lagged variable, to account for possible changes in the recovery-stress state of swimmers arising from TL exposure from the previous day [10]. None of the primary measures were related, significantly, with swim-training distance.

**TABLE 1:** Multilevel relationships between the baseline hormonal and psychological measures at the start (T1), middle (T2), and end (T3) of the 12-week training programme.

Time	Variables	Willingness to train	Stress	Sleep quality	Testosterone	Cortisol
T1	Willingness to train		0.10	0.08	0.22	-0.32
	Stress	0.14		0.17	0.03	-0.27
	Sleep quality	0.04	0.06		-0.20	0.12
	Testosterone	-0.03	0.25	-0.08		0.45*
	Cortisol	-0.26	0.25	-0.19	-0.07	

T2	Willingness to train		-0.28	-0.14	0.21	-0.21
	Stress	-0.34		-0.19	0.12	0.02
	Sleep quality	0.19	0.12		0.11	-0.07
	Testosterone	-0.18	-0.29	0.40		0.21
	Cortisol	0.04	-0.02	-0.10	0.31	

T3	Willingness to train		-0.19	0.14	-0.10	0.03
	Stress	0.16		0.39*	-0.11	0.05
	Sleep quality	0.06	-0.15		-0.12	-0.04
	Testosterone	0.30	0.18	-0.08		0.13
	Cortisol	0.31	-0.13	0.19	-0.25	

*Note:* Values above the diagonal at each time point are within-person correlations for male swimmers; values below the diagonal at each time point are within-person correlations for female swimmers.

* Significant correlation *p* < 0.05

## DISCUSSION

This study tracked hormonal and psychological adaptations in highly-trained swimmers across a 12-week training period, as they progressed towards the first major competition of the year. Contrary to our first hypothesis, training did not affect the salivary cortisol and testosterone measures, both baseline and exercise responses, willingness to train, and sleep quality. Only stress responded to training, decreasing at T2 and T3 (vs. T1) in female swimmers. No significant hormone-psychological relationships transpired, as per our second hypothesis, but some linkages (within each component) were seen in male swimmers at different time points.

For both male and female swimmers, baseline testosterone and cortisol concentrations did not change over 12 weeks of training, despite the incremental decrease in TL from T1 to T3. This finding is consistent with swimming training studies of varying duration [8, 9, 10, 11, 15, 20]. As part explanation, monitoring began four weeks after initial training, so any early-phase changes at T1 (our reference point) could affect subsequent adaptations to training. Emphasizing this point, male swimmers showed a decrease in testosterone concentration across the season, relative to off-season values [3] that, we argue, represents a true baseline in androgen activity. It is noteworthy that capillary blood measures of cortisol and testosterone, taken from our cohort under the same training conditions, did rise slightly at T3 (vs. T1) [4]. This could be explained by the fact that salivary steroids do not merely mirror blood-free hormones, but are more complex constituents [30] and better indicators of hormone exposure at target tissue. The sex differences observed, where male swimmers had more testosterone and female swimmers more cortisol, is supported by research [3, 4, 31].

The exercise-induced responses of testosterone (-21% to -34%) and cortisol (-52% to -72%) both declined across an early-morning swim session, as reported by others [4, 31], but no sex or training effects were detected. Conversely, when employing an afternoon (4 pm) exercise assessment, another cohort of swimmers displayed a transient rise in cortisol (93%), testosterone (24%) and free testosterone (44%) concentrations, as well as a training-related testosterone response [13]. Interestingly, a psychological stressor applied in a similar epoch (4–6 pm) was also effective in acutely elevating cortisol (31%) and testosterone (20%) concentrations in elite swimmers [32]. We attribute these trajectories to the diurnal decline in steroid hormones [33], whereby higher hormonal values in the early morning (vs. afternoon or late evening) make it difficult to active a stress response with an external stimulus, due to a ceiling effect. Another important difference lies in the use of a strong standardized stressor (i.e., 15 × 200 m freestyle efforts with 20-s rest periods) [13] rather than a typical swim-training session, as per this study. Furthermore, our estimates were derived from an aggregate of the Monday and Friday sessions that differed in training distance and intensity.

The stress scores of female swimmers decreased at T2 and T3, relative to T1. This covariation between perceived stress and TL (both declining) is congruent with swimming research examining different dimensions of general, specific, and total stress [7, 10] or fatigue [5]. It is not clear why the female swimmers showed greater stress sensitivity, given that TL exposure at T1, T2, and T3 was almost identical among men and women. This divergency might reflect the younger age and/or lesser training experience of female than male swimmers (*p* < 0.05, all large effects), or perhaps psychopathological differences between male and female athletes [34]. Over the course of a swimming season (up to 25 weeks), however, an excessive accumulation of training-induced fatigue might promote greater general stress and emotional exhaustion, even after a tapering period [22]. Willingness to train and sleep quality were stable across all participants from T1 to T3 and thus, appear less sensitive to major shifts in swimming TL, as illustrated by recent work using a combination of qualitative and quantitative sleep measures [4]. Sport motivation, which is conceptually similar to our metric of willingness to train, was similarly stable over several weeks of training in rugby league players [18] and Paralympic swimmers [7].

Correlational testing revealed that day-to-day changes in cortisol and testosterone concentrations tended to rise and fall together, but only in men at T1. There is broad evidence of positive testosterone and cortisol relationships in athletes and non-athletes [35, 36, 37, 38]. This association seems counterintuitive given the assigned catabolic and anabolic roles of cortisol and testosterone, respectively [32, 39]. These hormones could work in a cooperative manner to maintain homeostasis, especially under stress [36], perhaps explaining why this linkage was limited to the high-volume training phase. In a related study on elite male swimmers [32], a stronger positive cortisol and testosterone relationship was seen under a stress (*r* = 0.37) versus control (*r* = 0.26) trial, although neither result was significant due to a small sample. Among male swimmers, daily changes in sleep quality and stress also tended to covary at T3, which again seems counterintuitive in terms of direction and timing, although reported elsewhere [40]. Speculatively, these outcomes could be viewed as a pre-training preparatory response, at least for male swimmers, given situational (e.g., training phase) or contextual cues (e.g., stress / sleep changes arising from a recent performance). The presence of these associations in men, but not women, might again be explained by differences in age, training experience, and psychopathological make-up, as discussed above.

Our results have practical implications for researchers and practitioners. First, we corroborate the use of a stepwise reduction in TL to optimize performance [21] in a swimming context. The induction of hormonal and psychological adaptations was not, of course, a prerequisite for realizing this goal. Our stress results also support suggestions that subjective (vs. objective) measures exhibit superior sensitivity to track chronic TLs [41] and this can be achieved rapidly, and conveniently, using a simple Likert scale. For studies targeting the acute hormonal response, we advocate using a standardized exercise stimulus and implementation in the early evening / late afternoon to eliminate diurnal influences. Although less important to endpoint objectives, the daily monitoring of hormones and psychological state might elucidate nuanced relationships that help prepare athletes physically and/or mentally immediately prior to training. Our testing procedures are a study strength, characterized by five consecutive days of sampling at T1, T2, and T3. Replicating this design, if time and resources allow, would enhance statistical power and improve the precision of model estimates [42].

The current findings must be balanced against inherent limitations. For example, the number of athletes recruited was relatively small, but this was offset somewhat by the structure of testing (i.e., 18 athletes × 15 days). As a further caveat, we did not include the small proportion of “dry-land” training in our models, but presumably this had no effect on the studied parameters, being a supplemental stimulus and one that only accounted for 7–10% of total training time. The lack of hormone-psychological correlations could also be attributed, in part, to the metrics employed. More detailed (up to 76 items) stress-recovery-motivational inventories have been used in literature [2, 7, 10], but time constraints in a real-world setting necessitated an abbreviated questionnaire. Another consideration in our correlational results (or lack thereof) is the limited sample size, once data were separated by sex and time. Finally, our focus on cortisol and testosterone does not preclude a role for other metabolic, enzymatic or cardiovascular factors [6, 20, 22]. In this regard, small wearable sensors for pool and dry-land activities, integrating on-body sampling with edge computing, could be used to improve physiological monitoring and predictive diagnostics in sport [43].

The longitudinal testing of female athletes presents other challenges for sport science. Some female athletes, particularly elite performers, have demonstrated menstrual cycle fluctuations in not only training motivation and competitive desire (i.e., higher around ovulation), but also baseline concentrations of testosterone and testosterone responsivity to acute stress [16, 17, 44]. Unfortunately, we were unable to control for menstrual-cycle timing in our study design. Another issue arises from possible oral contraceptive (OC) usage, which we did not assess, as this can lead to a depressed testosterone concentration (up to 61%) in women [45, 46]. Even so, this lowering of testosterone (via OC use) does not appear to affect performance among elite female athletes [45], with meta-analytic evidence also showing a trivial effect of OC use on the exercise performance of women more generally [47].

In conclusion, a cohort of highly-trained swimmers experienced little or no adaptive changes in the lead up to a major competition. Self-reported stress was the exception, which decreased along with TL in female (but not male) swimmers. Among male (not female) swimmers, the emergence of daily interrelationships between selected hormonal or psychological outputs could provide a new lens to assess pre-training preparation.
